# Deep Eutectic Solvents (DESs) for the Isolation of Willow Lignin (*Salix matsudana cv. Zhuliu*)

**DOI:** 10.3390/ijms18112266

**Published:** 2017-10-28

**Authors:** Tengfei Li, Gaojin Lyu, Yu Liu, Rui Lou, Lucian A. Lucia, Guihua Yang, Jiachuan Chen, Haroon A. M. Saeed

**Affiliations:** 1 Key Lab of Pulp and Paper Science and Technology of the Ministry of Education, Qilu University of Technology, Jinan 250353, Shandong, China; leetengfly@gmail.com (T.L.); lalucia@ncsu.edu (L.A.L.); ygh@qlu.edu.cn (G.Y.); chenjc@qlu.edu.cn (J.C.); haroonsaeed75@gmail.com (H.A.M.S.); 2 College of Mechanical and Electronic Engineering, Shaanxi University of Science and Technology, Xi’an 710021, Shaanxi, China; lourui@sust.edu.cn; 3 Department of Forest Biomaterials, North Carolina State University, Box 8005, Raleigh, NC 27695-8005, USA; 4 Center of Fibers, Papers and Recycling, Faculty of Textiles, University of Gezira, Box 20, Wad Medani 79371, Sudan

**Keywords:** deep eutectic solvents, DES-lignin, choline chloride, lactic acid

## Abstract

Deep eutectic solvents (DESs) are a potentially high-value lignin extraction methodology. DESs prepared from choline chloride (ChCl) and three hydrogen-bond donors (HBD)—lactic acid (Lac), glycerol, and urea—were evaluated for isolation of willow (*Salix matsudana cv. Zhuliu*) lignin. DESs types, mole ratio of ChCl to HBD, extraction temperature, and time on the fractionated DES-lignin yield demonstrated that the optimal DES-lignin yield (91.8 wt % based on the initial lignin in willow) with high purity of 94.5% can be reached at a ChCl-to-Lac molar ratio of 1:10, extraction temperature of 120 °C, and time of 12 h. Fourier transform infrared spectroscopy (FT-IR) , ^13^C-NMR, and ^31^P-NMR showed that willow lignin extracted by ChCl-Lac was mainly composed of syringyl and guaiacyl units. Serendipitously, a majority of the glucan in willow was preserved after ChCl-Lac treatment.

## 1. Introduction

Lignocellulosic biomass is comprised of cellulose, hemicelluloses, and lignin. These biopolymers are promising building blocks for the burgeoning bioeconomy [1,2,3]. Willow (*Salix matsudana cv. Zhuliu*) is a new willow variety in the *Salicaceae Salix* spps., with high planting density, fast growth, strong resistance, and high survival rate, that has been widely applied in wind prevention and sand fixation. Moreover, willow has a good potential for the pulp and paper industry because it is fast-growing, particularly in China [4,5].

Ionic liquid (IL) pretreatment of biomass has attracted broad scientific attention despite high costs and possible toxicity [6,7]. However, deep eutectic solvents (DESs), an alternative to ILs, are rapidly emerging [8]. DESs are ambient temperature ionic liquids composed of two or three ionic compounds (hydrogen bond donor and hydrogen bond receptor) capable of combinations to form a eutectic mixture [9,10]. Eutectics are made at a high melting point to display a melting point lower than that of each individual component. DESs exhibit analogous physico-chemical properties to ILs, but can be inexpensive and environmentally friendly [11,12,13,14]. Current research and development efforts have focused on the use of DESs for cellulose treatment from biomass [15]. Lignin is the most abundant renewable aromatic polymer on Earth, present in nearly all higher plants, which behooves the development of effective methods to extract it [16]. Its reactivity is primarily characterized by a large number of phenolic hydroxyls [17]. Previous studies have shown that DESs formed by choline chloride and phenol were conducive to the separation of phenolic compounds [18].

To date, there has not been a detailed investigation on applying DESs for extracting lignin from willow. In this work, three different DESs were used, and the effects of treatment time and temperature of the optimal DESs on extraction of lignin from willow was investigated. The lignin extracted from willow by DES treatment that was defined as DES-lignin was characterized by Fourier transform infrared spectroscopy (FT-IR), ^13^C-NMR, and ^31^P-NMR.

## 2. Results and Discussion

### 2.1. Effect of Different Deep Eutectic Solvents (DESs) and Temperture on the Extraction of Lignin from Willow

To investigate the effect of different DES and temperature on the extraction of willow lignin, three DES mixtures with the same mole ratio of choline chloride (ChCl) to three HBDs (molar ratio 1:2): glycerol (ChCl-Gly), urea (ChCl-U), and lactic acid (ChCl-Lac) were prepared. The treatment of willow by each DES was conducted at four different temperatures: 90, 100, 110, and 120 °C over 6 h.

**Figure 1:**
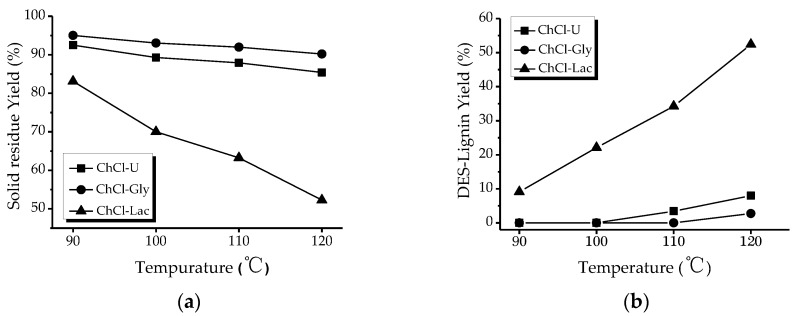
Effect of deep eutectic solvents (DESs) type and temperature on the solid residue yield (**a**) and DES-lignin yield (**b**) (molar ratio of choline chloride (ChCl) to hydrogen-bond donors (HBD) was 1:2, and the treatment time was 6 h).

The solid residue and DES-Lignin yield are shown in Figure 1. It was shown that a negligible amount of lignin was removed by ChCl-Gly and ChCl-U treatment. Nonetheless, it was remarkable to find that ChCl-Lac treatment had a pronounced effect on lignin isolation which increased with increasing treatment severity. It had been reported that DESs could promote the cleavage of ether bonds and, thus, favorable to lignin degradation and/or facilitate lignin extraction from wood fibers [19]. In addition, under hydrolysis or hydrothermal conditions, hemicellulose degraded and removed from willow would assist in the extraction of lignin. Several studies have illustrated that DESs with high H-bond accepting ability and polarity would contribute to dissolving lignin [20]. Compared to glycerol and urea, lactic acid had more suitable solution parameter values according to Hansen’s theory of dissolution [21]. All these showed that the ChCl-Lac treatment was found to be much more suitable compared to ChCl-Gly and ChCl-U. More specifically, 52.43% based on the initial lignin could be obtained at 120 °C. This latter result is in keeping with previous studies showing that the solubility of lignin in DESs increases with temperature [22].

### 2.2. Effect of Different Mole Ratio of Choline Chloride (ChCl) to Lactic Acid (Lac) on the Extraction of Lignin from Willow

**Figure 2:**
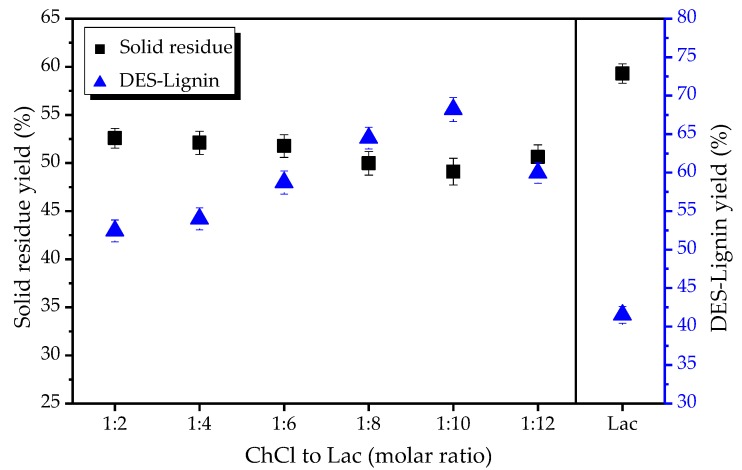
Effect of different mole ratio of choline chloride (ChCl) to lactic acid (Lac), as well as Lac which was not formed DES with ChCl on the solid residue yield and DES-Lignin yield. The treatment conditions: temperature, 120 °C; time: 6 h.

Six different mole ratios of ChCl to Lac (molar ratio 1:2, 1:4, 1:6, 1:8, 1:10, 1:12) were carried out and compared at 120 °C and 6 h. As a control, pure lactic acid (Lac) treatment on the removal of willow components was also studied. In Figure 2, the soluble part of willow increased with the increase of lactic acid in DESs, while the DES-Lignin yield reached a maximum at a mole ratio of 1:10. At an excessive lactic acid ratio, the ChCl-Lac molar ratio of 1:12 led to a decrease in lignin yield. Furthermore, pure lactic acid treatment resulted in fewer chemical components removed compared to DESs, as shown by 41.52% of the initial lignin extraction.

### 2.3. Effect of the DESs Treatment Time on Extraction of Lignin from Willow

**Figure 3:**
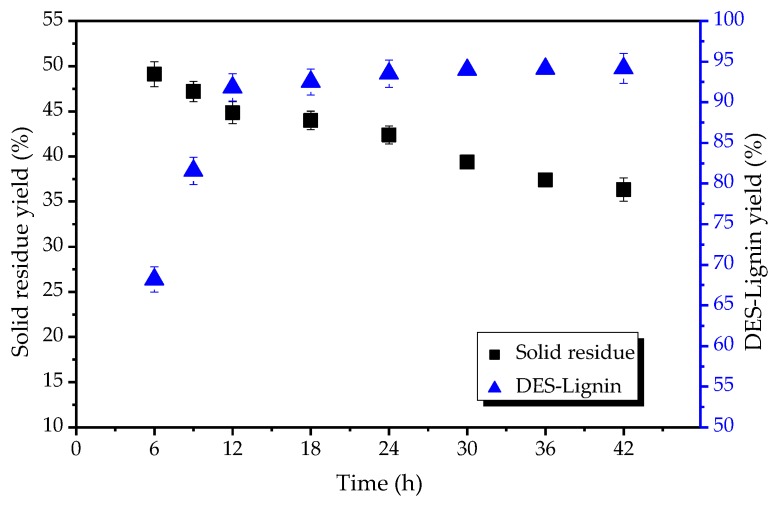
Solid residue yield and DES-Lignin yield varying with treatment time after ChCl-Lac (molar ratio 1:10) treatment at a treatment temperature of 120 °C.

ChCl-Lac with a molar ratio of 1:10 was among the most effective DESs for willow treatment. The amount of lignin extracted at this latter ratio increased significantly with increasing treatment time from 6 h to 12 h (Figure 3). However, a negligible DES-Lignin yield was observed when the treatment time was greater than 12 h. For example, when the treatment time was extended to 42 h, DES-Lignin yield was 94.2%, sightly more than the DES-Lignin yield at 12 h (91.8%). However, the solid residue yield was reduced as the treatment time prolonged, indicating the carbohydrate components dissolved and/or degraded markedly. Thus, a treatment time of 12 h was sufficient for high lignin yield and avoiding carbohydrate degradation.

### 2.4. Chemical Composition Analysis of Solid Residue and Dissolved Carbohydrates in the Mixtures

**Figure 4:**
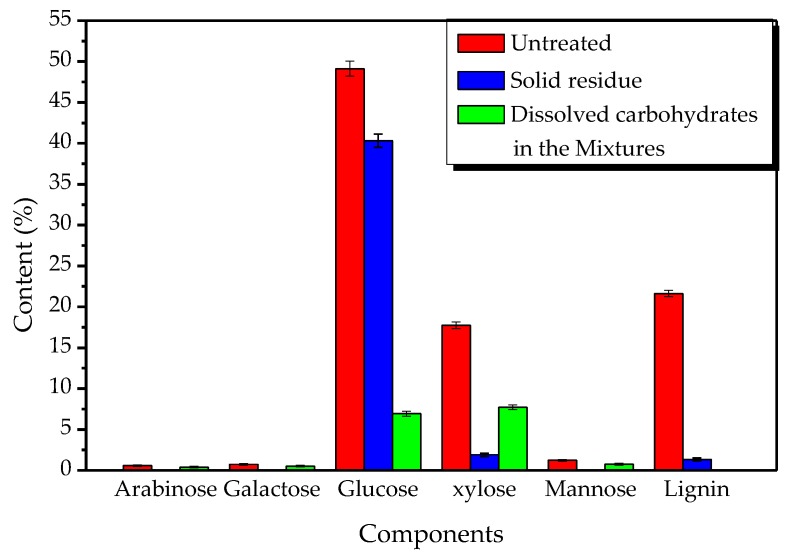
The composition of solid residue and dissolved carbohydrates in the mixtures after choline chloride-lactic acid (ChCl-Lac, molar ratio 1:10) treatment at 120 °C for 12 h (on dry basis of the initial sample).

The chemical composition of the solid residue and dissolved carbohydrates after ChCl-Lac (1:10) treatment at 120 °C for 12 h was characterized for sugars and lignin (Figure 4, on the dry basis of the initial sample). The content of each chemical composition in the final solid product and/or dissolved in the mixture phase, which was expressed as a percentage of the initial component, is further presented in Table 1. It was very intriguing to find that 82.07% of the initial glucose was preserved in the final solid residue while only 6.20% of the initial lignin was retained, suggesting that a large amount of lignin was extracted from willow and the vast majority of glucan in the initial sample was well preserved after DESs treatment. Indeed, 73.52% of xylose, 70.69% of arabinose, 72.00% of galactose, and 81.30% of mannose (as a percentage of the initial component) were in the mixtures, whereas only 10.77% of the initial xylose was retained in the solid residue, suggesting the majority of xylan, arabinan, galactan, and mannan were removed in the ChCl-Lac (1:10) treatment. This may be because xylan, arabinan, galactan, and mannan are susceptible to thermal degradation and removal under the hydrolysis [23,24]. Interestingly, a fraction of 91.8% of the initial lignin was extracted by ChCl-Lac (1:10) treatment at 120 °C for 12 h. This shows that DESs was an effective solvent for extracting lignin. Compared with the traditional ionic liquid or other organic solvents [25,26], more than 90% of the initial lignin could be extracted under moderate reaction conditions while, at the same time, a vast majority of glucan was not degraded and well preserved.

### 2.5. Lignin Purity Analysis

To investigate the purity of lignin using ChCl-Lac (1:10) treatment at 120 °C for 12 h, the acid insoluble lignin (AIL), acid soluble lignin (ASL), sugars, and ash content in DES-Lignin were measured (Table 2). Only 0.21% of glucose, 0.15% of xylose and 0.51% of ash were present, whereas the DES-Lignin isolated from ChCl-Lac (1:10) treatment at 120 °C for 12 h had a purity of 94.46%.

### 2.6. Structure Characterization of DES-Lignin

#### 2.6.1. Fourier Transform Infrared Spectroscopy (FT-IR)

**Figure 5:**
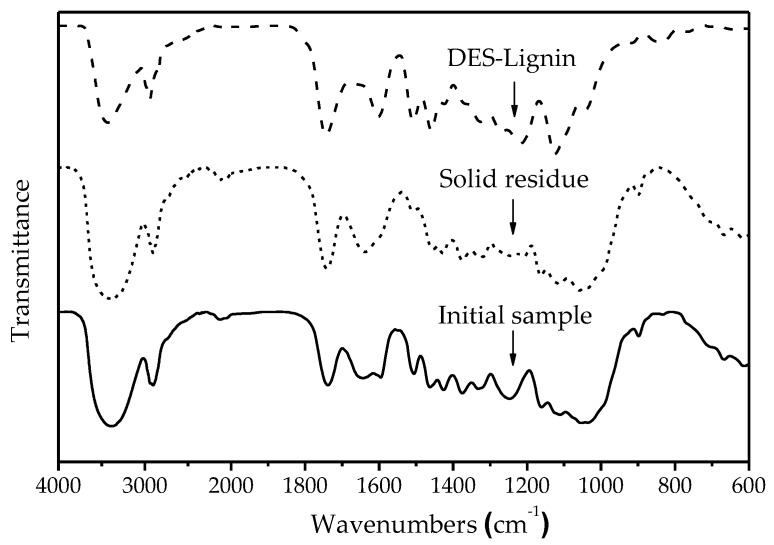
Fourier transform infrared spectroscopy (FT-IR) spectra of the initial sample, solid residue, and DES-Lignin.

The FT-IR spectra of the initial sample, solid residue, and DES-lignin are shown in Figure 5, and the signal assignments are listed in Table 3. Compared with the initial sample, the intensity of the bands at 1600, 1510, 1270, 1120, and 835 cm^−1^ in the solid residue were significantly reduced, which indicated that most of the lignin was removed from willow after ChCl-Lac (1:10) treatment [27,28,29].

The bands at 1600 and 1510 cm^−1^ indicated that the benzene ring skeleton of lignin was left intact. In addition, the higher intensity of the bands at 1325, 1220, and 1120 cm^−1^ indicated that the syringyl unit played a dominant role. In contrast, the lower intensity of the bands at 1270 and 1035 cm^−1^, and the absence of the band at 1156 cm^−1^ suggested that a reduced level of guaiacyl units and a negligible amount of *p*-hydroxyphenyl units were in the DES-Lignin. The bands at 1426, 1373, 1164, and 1056 cm^−1^ were absent, suggesting most of the carbohydrates were not present in the DES-Lignin. However, a negligible amount of hemicellulose present in DES-Lignin due to the existence of the band at 1710 cm^−1^ was consistent with the results of previous lignin purity analyses [30,31].

#### 2.6.2. ^13^C-NMR

**Figure 6:**
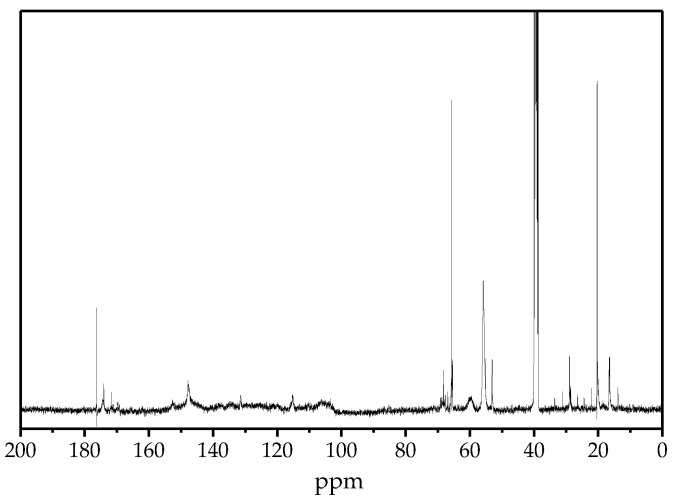
^13^C NMR spectra of DES-Lignin.

The ^13^C-NMR spectra of DES-Lignin are presented in Figure 6. The positions of signals are listed in Table 4 as assigned previously [32,33]. The lack of signals between 90 and 102 ppm confirms the absence of carbohydrates in DES-Lignin. The existence of the β-O-4 ether linkage and β-β linkage were indicated by signals at 57 to 61 ppm, 52 to 54 ppm [34]. Another striking observation is the high level of phenolic hydroxyl groups (171 to 174 ppm) in DES-Lignin. Moreover, the presence of signals at 103 to 108 ppm, 131.0 to 132.1 ppm and 150.3 to 153 ppm suggested that syringyl units were present in DES-Lignin. However, only one signal located at 146.2 to 149.0 ppm associated with the guaiacyl indicated a low amount of guaiacyl. A non-trivial signal of 114.2 to 116.0 ppm indicated that *p*-coumaric acid was in DES-Lignin [35].

#### 2.6.3. ^31^P-NMR

^31^P-NMR was further used for the quantitation of hydroxyl groups in DES-lignin, and the functional group content from quantitative ^31^P-NMR is listed in Table 5 as assigned previously [36]. As shown in Table 5, the DES-Lignin extracted from willow could be recognized as GHS lignin, and the G:H:S ratio was 2.59:1:4.73, indicating that the syringyl unit was dominant in DES-Lignin, which was consistent with the typical structure of hardwood lignin. It was interesting that the content of the total phenol hydroxyl group in DES-Lignin (0.915 mmol·g^−1^) was higher than that of poplar alkali lignin (AL) or other biorefinery lignins [37], suggesting that the DES-lignin had a higher potential reactivity.

## 3. Materials and Methods

### 3.1. Materials

The five year old raw material of willow (*Salix matsudana cv. Zhuliu*) was harvested and sawed in a forestry center in Dingxi, Gansu Province, China. The representative sample of the sawed willow trunk (bark and leaves excluded, a length of 1.50 m, and a diameter cross-section of 22 cm, approximately 25 kg) was collected and air-dried at ambient temperature in lab for one month. The air-dried sample was cut to 3–5 cm in size and a part of the sample was ground in a star mill. The 40 to 80 mesh fractions were used as the raw material for composition analysis and the subsequent DESs treatment. The chemical composition of willow (wt %) was determined according to the Laboratory Analytical Procedure for Biomass provided by NREL [38] and the results are presented in Table 6. Choline chloride, lactic acid, glycerol, and urea were purchased from Sinopharm Chemical Reagent Co., Ltd. (Shanghai, China).

### 3.2. DESs Preparation

Three different DESs were prepared: choline chloride to glycerol (ChCl-Gly) (molar ratio 1:2), choline chloride to urea (ChCl-U) (molar ratio 1:2), and choline chloride to lactic acid (ChCl-Lac) (molar ratio 1:2, 1:4, 1:6, 1:8, 1:10, 1:12). The solutions were stirred in an oil bath at 60 °C to form a homogeneous liquid without of any solid particles. The mixture was cooled down and stored in a desiccator for further use.

### 3.3. DESs Treatment of Biomass (Willow)

Prior to DES treatment, the sample was Soxhlet-extracted with the mixture of benzene and 95% ethanol for 8 h to remove extractives. After that the sample was dried in the vacuum oven at 60 °C for 12 h. For each run, 2.5 g of extractive free sample was added into DESs solvent in reaction vessels with the solvent to solid ratio of 30:1 (*g*:*g*). The vessel was then heated by oil bath to the desired temperature to start the reaction. The effect of HBD types (lactic acid, urea, glyreol), the molar ratio of choline chloride to lactic acid (1:2, 1:4, 1:6, 1:8, 1:10, 1:12), the reaction temperatures (90–120 °C), and the reaction time (6–42 h) were examined. Each run was conducted in at least triplicate until the data had good reproducibility.

**Figure 7:**
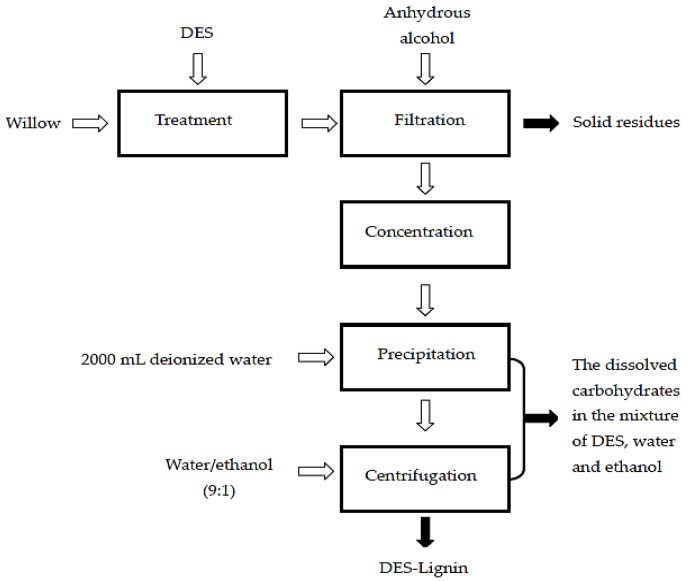
Flow diagram of willow treatment with DESs.

After treatment, the solid residue and DES soluble fractions were separated by filtration using a glass crucible with anhydrous alcohol washing until the solid fraction recovered was a lighter shade; the solid residue and the glass crucible were dried in a vacuum oven. The filtrate was concentrated and added to deionized (DI) water (2000 mL) to precipitated lignin. After 24 h, lignin was collected after centrifugation, and washed with a water/ethanol mixture (9:1) [19]. The DES-Lignin (the lignin extracted from willow by DESs treatment) was obtained by vacuum freeze-drying. The whole process is shown in Figure 7.

Solid residue yield was calculated as follows:

(1) $$\frac{\mathrm{Mass}\;\mathrm{of}\;\mathrm{the}\;\mathrm{solid}\;\mathrm{residue}\;\mathrm{after}\;\mathrm{DESs}\;\mathrm{treatment}}{\mathrm{Mass}\;\mathrm{of}\;\mathrm{the}\;\mathrm{initial}\;\mathrm{dry}\;\mathrm{sample}}$$

DES-Lignin yield was calculated as follows:

(2) $$\frac{\mathrm{Mass}\;\mathrm{of}\;\mathrm{the}\;\mathrm{lignin}\;\mathrm{extracted}\;\mathrm{by}\;\mathrm{DESs}\;\mathrm{treatment}}{\mathrm{Mass}\;\mathrm{of}\;\mathrm{the}\;\mathrm{lignin}\;\mathrm{in}\;\mathrm{the}\;\mathrm{initial}\;\mathrm{sample}}$$

### 3.4. Chemical Composition of Solid Residue, DES-Lignin, and the Dissolved Carbohydrates in the Mixtures

The chemical composition of the solid residues and DES-Lignin were analyzed according to the NREL methods [38]. Dissolved carbohydrates were quantitatively tested by using acid hydrolysis. Firstly, stoichiometric 72 wt % sulfuric acid was added to the mixture of DES and DI water so that the final acid concentration was 4 wt %. Then the solution was hydrolyzed for 60 min at 121 °C in an oil bath [39]. The hydrolyzed mixture liquid was filtered (0.22 μm) and, subsequently, the liquid was used for the determination of monomeric sugars. The monomeric sugars were quantitated by using an ion chromatography system (ICS-5000, Thermo Fisher, Sunnyvale, CA, USA) [40,41].

For convenient comparison, the content of monosaccharides was calculated as follows:

(3) $$\frac{\mathrm{Mass}\;\mathrm{of}\;\mathrm{the}\;\mathrm{monosaccharide}\;\mathrm{in}\;\mathrm{the}\;\mathrm{solid}\;\mathrm{residue}\;\mathrm{or}\;\mathrm{the}\;\mathrm{mixtures}\;}{\mathrm{Mass}\;\mathrm{of}\;\mathrm{the}\;\mathrm{initial}\;\mathrm{component}}$$

### 3.5. Structure Characterization of the DES-Lignin

#### 3.5.1. FT-IR Spectroscopy

The DES-Lignin was characterized by FT-IR (ALPHA, Bruker, Karlsruhe, Germany). The dried samples were embedded in KBr pellets at concentrations of ~1 mg/100 mg KBr. All spectra were recorded in absorption band mode over the range of 4000 to 600 cm^−1^.

#### 3.5.2. ^13^C-NMR

The ^13^C-NMR spectra were recorded on a 400 MHz Bruker AVANCE III NMR spectrometer (Karlsruhe, Germany). Samples of 60 mg were completely dissolved in 500 μL of dimethyl sulfoxide (DMSO-*d*_6_). The chemical shifts were calibrated relative to the signals of DMSO-*d*_6_, which was used as an internal standard at 39.5 ppm for the ^13^C-NMR spectra. The acquisition time was 1.4 s and the relaxation time was 1.7 s.

#### 3.5.3. ^31^P-NMR

The ^31^P-NMR spectra were recorded on a 400 MHz Bruker AVANCE III NMR spectrometer (Karlsruhe, Germany). Twenty-five milligrams of sample were completely dissolved in 400 μL of anhydrous pyridine and deuterated chloroform (1.6:1, *v*/*v*). Then 150 μL of cyclohexanol/chromium(III) acetylacetonate solution (4 mg·mL^−1^/3.6 mg·mL^−1^ in anhydrous pyridine and deuterated chloroform 1.6:1, *v*/*v*) was added as an internal standard and relaxation reagent, respectively. The mixture was reacted with 75 μL of phosphating reagent (2-chloro-4,4,5,5-tetramethyl-1,3,2-dioxaphospholane-tetramethyl-1,3,2-dioxaphospholane, TMDP) for about 5 min and then was transferred into a 5 mm NMR tube for subsequent NMR test.

## 4. Conclusions

Deep eutectic solvents (DESs) were shown to be an effective method for the isolation of lignin from willow lignocellulosic biomass (*Salix matsudana cv. Zhuliu*) at high yield and high purity. Reaction conditions, including reaction time, temperature, and HBD types and dosage, were all shown to be important for extraction. DESs formed by choline chloride and lactic acid (molar ratio = 1:10) at 120 °C and 12 h were appropriate and resulted in 91.82% DES-lignin yield. Extracted DES-lignin displayed high purity (94.46%) with unique structural properties as indicated by FT-IR spectroscopy, ^13^C-NMR, and ^31^P-NMR. Moreover, more than 80% of the initial glucan was recovered as a solid residue for further enzymatic hydrolysis or chemical conversion.

## Figures and Tables

**Table 1:** The chemical composition of the solid residue and dissolved in the mixtures after choline chloride-lactic acid (ChCl-Lac) (1:10) treatment at 120 °C for 12 h (as a percentage of the initial component).

Component	Arabinose (%)	Galactose (%)	Glucose (%)	Xylose (%)	Mannose (%)	Lignin (%)
Solid residue	-	-	82.07	10.77	-	6.20
Dissolved in the mixtures	70.69	72.00	14.13	73.52	81.3	91.8 *

* The amount of lignin extracted by deep eutectic solvents (DESs).

**Table 2:** Purity analysis of the lignin extracted from willow by ChCl-Lac (1:10) treatment at 120 °C for 12 h.

Component	Amount (%)
Acid insoluble lignin (AIL)	92.57 ± 0.7
Acid soluble lignin (ASL)	1.89 ± 0.1
Glucose	0.21 ± 0.05
Xylose	0.15 ± 0.03
Arabinose	-
Galactose	-
Mannose	-
Ash	0.51 ± 0.1
Lignin purity *	94.46 ± 0.8

* Lignin purity is calculated from the sum up of AIL (%) and ASL (%) of willow.

**Table 3:** Assignment of Fourier transform infrared spectroscopy (FT-IR)spectra of the initial sample, solid residue, and DES-Lignin.

Wavenumbers (cm^−1^)	Assignment (Bond)	DES-Lignin	Solid Residue	Initial Sample
3410	O–H stretching vibration	3410	3410	3410
2924	C–H stretching vibration in methyl, methylene	2924	2924	2924
1710	C=O stretching vibration	1710	1710	1710
1600, 1510	Aromatic ring skeleton vibration	1600, 1510	-	1600, 1510
1460	C–H deformation vibration in –CH_2_–	1460	1460	1460
1426	C–H bending vibration in –CH_2_– of cellulose	-	1426	1426
1373	C–H bending vibration of aliphatic compounds	-	1373	1373
1325, 1220	C–O stretching vibration of syringyl units	1325, 1220	1325	1325, 1220
1270	C–O stretching vibration of guaiacyl units	1270	-	1270
1164	C–O–C symmetrical stretching vibration in carbohydrate	-	1164	1164
1120/835	C–H stretching vibration of syringyl units	1120/835	-	1120/835
1056	C=O stretching vibration in carbohydrate	-	1056	1056
1035	C–H bending vibration of guaiacyl units	1035	1035	1035

**Table 4:** ^13^C NMR analysis of DES-Lignin.

Chemical Shift (ppm)	Assignment
171–174	Phenolic OH
169–171	Aliphatic OH
150.3–153.0	C_Ar_ in etherified syringyl units
146.2–149.0	C_Ar_ in non-etherified guaiacyl units
131.0–132.1	C_Ar_ in non-etherified syringyl units
114.2–116.0	C_Ar_ in *p*-coumaric acid ester
103–108	C_Ar_ in syringyl units
67–71	C_α_ in β-O-4
57–61	C_γ_ in β-O-4
54–57	CH_3_O
52–54	C_β_ in β-β or β-5

**Table 5:** ^31^P NMR analysis of DES-Lignin.

Chemical Shift (ppm)	Assignment	Content (mmol·g^−1^)
133.8–135.0	Carboxyl	0.182
137.0–138.4	p-phenol hydroxyl (H)	0.110
138.4–140.2	Guaiacyl phenol hydroxyl (G)	0.285
142.4–143.7	Syringyl phenol hydroxyl (S)	0.520
-	G:H:S	2.59:1:4.73
140.2–142.4, 143.7–144.4	Condensation phenol hydroxyl	0.539
145.2–150.1	Aliphatic hydroxyl	0.606
-	Total phenol hydroxyl	0.915
-	Total hydroxyl	2.06

**Table 6:** Composition analysis of willow.

Composition	Willow (wt %)
Arabinose	0.58
Galactose	0.75
Glucose	49.13
Xylose	17.74
Mannose	1.24
Total lignin	21.63
Acid insoluble lignin	18.63
Acid soluble lignin	3.00
Benzene-alcohol extractive	3.10
Ash	0.91
